# Themes Surrounding COVID-19 and Its Infodemic: Qualitative Analysis of the COVID-19 Discussion on the Multidisciplinary Healthcare Information for All Health Forum

**DOI:** 10.2196/30167

**Published:** 2022-05-11

**Authors:** Rakshith Gangireddy, Stuti Chakraborty, Neil Pakenham-Walsh, Branavan Nagarajan, Prerna Krishan, Richard McGuire, Gladson Vaghela, Abi Sriharan

**Affiliations:** 1 Institute of Health Policy, Management, and Evaluation University of Toronto Toronto, ON Canada; 2 Occupational Therapy Rehabilitation Institute Christian Medical College Vellore India; 3 Global Healthcare Information Network Charlbury United Kingdom; 4 Global Health Academy University of Edinburgh Edinburgh United Kingdom; 5 Gujarat Medical Education & Research Society Medical College Gandhinagar India

**Keywords:** infodemic, infodemiology, COVID-19, pandemic, misinformation, health information, theme, public health, qualitative study, global health

## Abstract

**Background:**

Healthcare Information for All (HIFA) is a multidisciplinary global campaign consisting of more than 20,000 members worldwide committed to improving the availability and use of health care information in low- and middle-income countries (LMICs). During the COVID-19 pandemic, online HIFA forums saw a tremendous amount of discussion regarding the lack of information about COVID-19, the spread of misinformation, and the pandemic’s impact on different communities.

**Objective:**

This study aims to analyze the themes and perspectives shared in the COVID-19 discussion on English HIFA forums.

**Methods:**

Over a period of 8 months, a qualitative thematic content analysis of the COVID-19 discussion on English HIFA forums was conducted. In total, 865 posts between January 24 and October 31, 2020, from 246 unique study participants were included and analyzed.

**Results:**

In total, 6 major themes were identified: infodemic, health system, digital health literacy, economic consequences, marginalized peoples, and mental health. The geographical distribution of study participants involved in the discussion spanned across 46 different countries in every continent except Antarctica. Study participants’ professions included public health workers, health care providers, and researchers, among others. Study participants’ affiliation included nongovernment organizations (NGOs), commercial organizations, academic institutions, the United Nations (UN), the World Health Organization (WHO), and others.

**Conclusions:**

The themes that emerged from this analysis highlight personal recounts, reflections, suggestions, and evidence around addressing COVID-19 related misinformation and might also help to understand the timeline of information evolution, focus, and needs surrounding the COVID-19 pandemic.

## Introduction

Health systems fighting the COVID-19 pandemic worldwide are facing a secondary challenge of having to address the accompanying infodemic, defined by the World Health Organization (WHO) as an overabundance of information—some accurate and some not—that makes it hard for people to find trustworthy sources and reliable guidance when they need it [1].

Infodemics are a rapidly rising global health issue. The modern digitized world has amplified various information channels, such as social media and online forums, allowing them to spread information much faster and further due to the availability and accessibility of technology as well as a lack of traditional quality control [2,3]. The resulting increase in health-related overabundance of information and misinformation hinders policy makers and health care workers from finding trustworthy sources and reliable guidance when they need it [4]. Furthermore, infodemics have been linked to negative health consequences, as showcased by the measles outbreaks in countries such as the United Kingdom, the United States, Germany, and Italy as a result of vaccine hesitancy fueled by misinformation [5,6]. Likewise, infodemics have also led to violence and distrust, as seen by the targeted attacks on health care workers during the 2019 Ebola outbreak in the Democratic Republic of Congo [7]. Thus, the current infodemic surrounding COVID-19 is not a novel phenomenon but part of a global public health trend that has been significantly growing over the past few years.

Many recent studies have attempted to characterize the infodemic and its predisposing factors. In rapidly evolving situations, such as the COVID-19 pandemic, an explosive amount of new information is generated and researchers, policy makers, journalists, and ordinary citizens are unable to keep up with the evolving facts [8]. In addition, incoherent public health messaging and reversals in recommendations cause distrust in governments and health authorities [9]. Furthermore, people prefer and tend to accept information that confirms and is consistent with their preexisting attitudes and beliefs even if that information is not based in evidence [10]. Poor health literacy shapes interpretation of information. Poor health journalism by traditional forms of media is also found to be a factor [11]. Lastly, the lack of accurate and reliable scientific knowledge closer to the broader population allows for unverified information to fill the gaps left behind [12].

To effectively address the COVID-19 pandemic and future public health emergencies, infodemics must be understood and managed. WHO established the Information Network for Epidemics (EPI-WIN) [13] to counter the COVID-19 infodemic and mitigate its side effects. The United Nations (UN) launched a portal for the public to access reliable and up-to-date COVID-19 information through its Verified initiative [14]. Similarly, the US Centers for Disease Control and Prevention (CDC) created a series called “COVID-19 Science Update” to aid public health professionals’ response to COVID-19 [15]. Health authorities worldwide are working closely with online platforms, including Facebook, Google, Twitter, and YouTube, to provide and highlight evidence-based information [2]. Ultimately, the right message at the right time from the right messenger through the right medium can save lives [13].

Healthcare Information for All (HIFA) is a multidisciplinary global campaign consisting of more than 20,000 members worldwide committed to improve the availability and use of health care information in low- and middle-income countries (LMICs) [16]. Sponsored by the University of Edinburgh, HIFA is primarily based around virtual communities of practice that allow for the discussion of different health care topics with a focus on information needs. The forums use reader-focused moderation to create an organic atmosphere that allows for topics to emerge that are of interest to the forum members [17].

COVID-19 and the infodemic surrounding it have become a major discussion theme on the HIFA forums. The first post about COVID-19 on HIFA was published on January 24, 2020. Since then, over 1000 posts have been created on the topic—surpassing the number of posts made about any other topic previously on the forums. It was hypothesized that this discussion could provide an understanding of the information needs that surround the COVID-19 pandemic, particularly in LMICs, and what may be contributing to the infodemic.

This analysis aims to contribute to the global effort to track, understand, and respond to the infodemic surrounding the COVID-19 pandemic by identifying themes and perspectives shared by members on the HIFA forums.

## Methods

### Data Analysis

**Figure 1:**
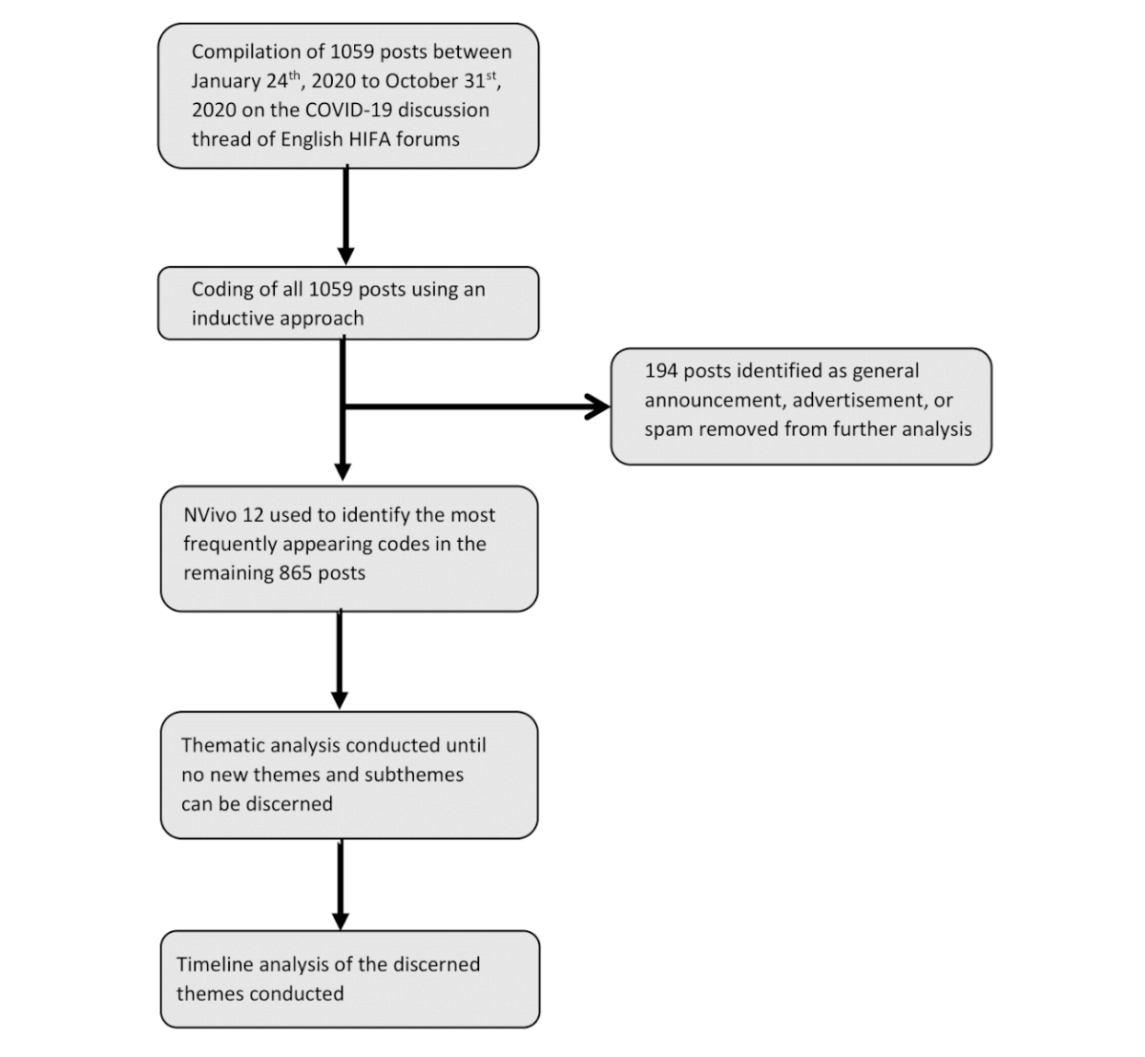
Schematic of the qualitative study analysis method.

A thematic content analysis of the COVID-19 discussion on English HIFA forums was conducted (Figure 1). The full text of all 1059 COVID-19-related discussion posts between January 24 and October 31, 2020, on the forums was collected, and each post was coded by 4 team members (authors RG, SC, RM, and PK) using an inductive coding approach. We kept track of the codes on a common document to reduce redundancy and ensure intercoder reliability. Codes included geographic locations (ie, countries, continents), populations (ie, refugees, children, migrant workers), and topics of the post (ie, mental health, use of chloroquine, herd immunity). Of the original 1059 posts, 194 (18.32%) were removed because they were found to be general announcements, spam messages, and advertisements that did not contribute meaningfully to the COVID-19 discussion. The qualitative analysis software NVivo 12 (QSR International) [18] was then used to identify the most frequently appearing codes in the remaining 865 (81.68%) posts and develop themes and subthemes [19] using a grounded theory approach until no new themes were discerned.

A timeline analysis of the posts divided by month was also conducted. The 865 posts were divided according to the months in which they were posted. Within each month, the 20 most frequently mentioned words, excluding articles and conjunctions (ie, the, of, because) and similar nonmeaningful words, were acquired using NVivo 12. These words were then used to determine the most common topics for each month of the COVID-19 discussion on the HIFA forums.

A secondary analysis was conducted on the profile data of all HIFA members who contributed to the COVID-19 discussion in order to understand their backgrounds as study participants. This analysis included the members' location of residence, their profession, and their affiliation. The professions were broadly categorized into researchers, health care professionals, public health workers, information providers, and others. Similarly, the affiliations were broadly categorized into government, WHO, UN, commercial organizations, nonprofit nongovernment organizations (NGOs), academia, and others.

### Ethical Considerations

Prior to the study being undertaken, a formal message was sent to members of the HIFA forums, introducing its purpose and obtaining implied consent. Formal consent was not obtained from each individual member as all content on the HIFA forums, including the discussion posts and member data, is publicly shared information. The study was assessed by the researchers to be low risk. Identifying data, such as names and addresses, that can be reasonably used to identify individuals were removed from the posts during the initial coding process to ensure individual member confidentiality.

## Results

### Study Participants

**Figure 2:**
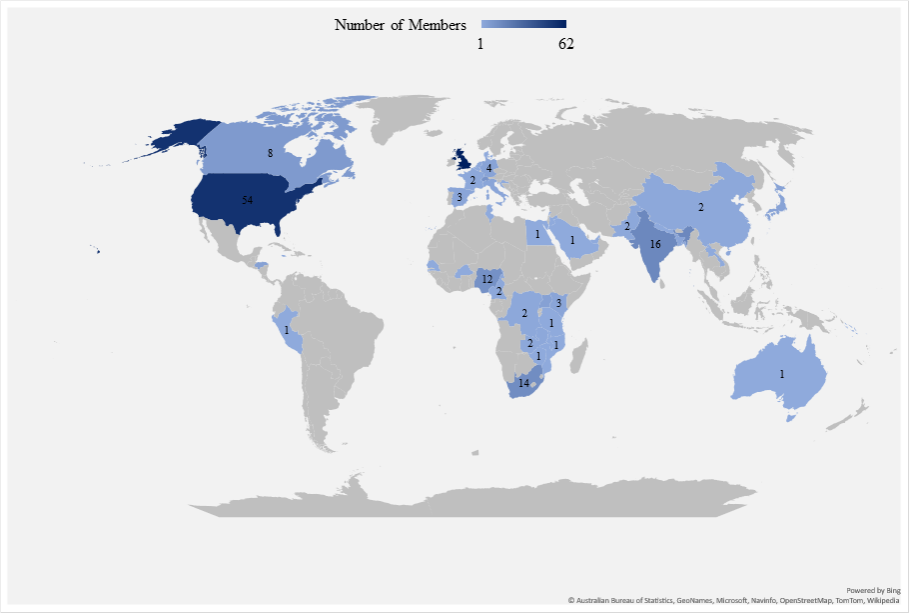
Geographical distribution of the study participants. In total, 246 members across 46 countries from every continent except Antarctica participated in the COVID-19 discussion. The United Kingdom had the greatest number of study participants at 62 (25.2%), with the United States being second with 54 (22%) participants and India being third with 16 (6.5%) participants.

In total, 246 members across 46 different countries participated in the discussion. The geographical data (Figure 2) revealed that the top 3 countries in descending order are the United Kingdom (n=62, 25.2%), the United States (n=54, 22%), and India (n=16, 6.5%). Every continent except Antarctica was represented, with the main regions being Europe, North America, and Africa.

**Figure 3:**
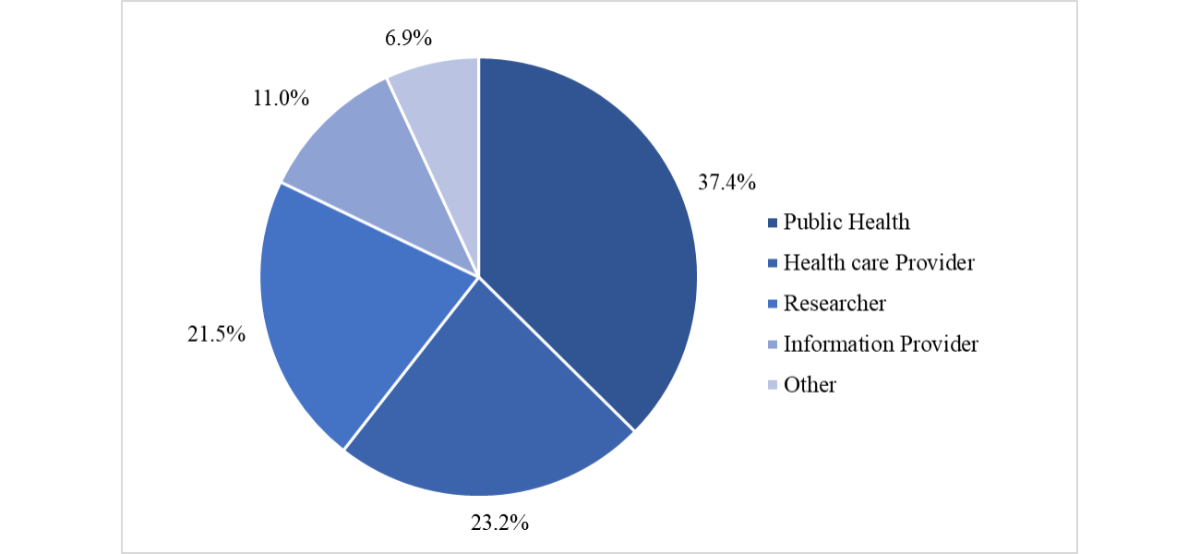
Categories of professions represented by the study participants. Most of the study participants fell into the category of public health, which included public health officials, policy makers, and consultants. Health care providers included physicians, nurses, and CHWs. The category of information provider included librarians, editors and associate editors of journals, and communications specialists. The other category included students, volunteers, and retired members. CHW: community health worker.

A significant number of HIFA members’ professions (Figure 3) could be categorized as public health workers (eg, public health registrars and consultants at global health organizations), who numbered 92 (37.4%). Health care providers, such as physicians, nurses, and community health workers (CHWs), and researchers holding academic positions made up the second and third categories, with 57 (23.2%) and 53 (21.5%) members, respectively.

**Figure 4:**
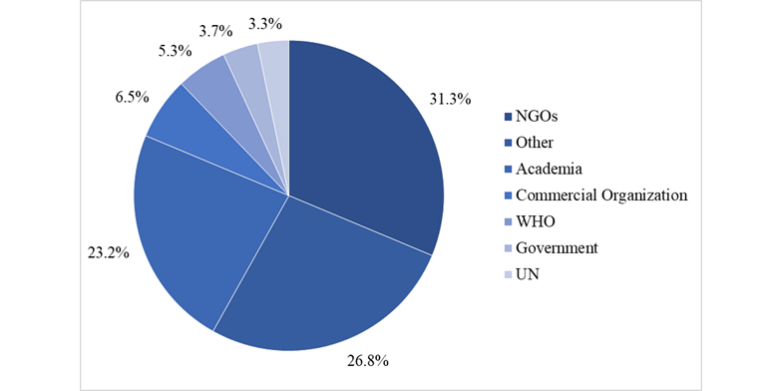
Affiliations of the study participants. The largest affiliation was nonprofit local NGOs with 77 (31.3%) members. The other category of affiliations included independent professionals, volunteers, and retired members. NGO: nongovernment organization; UN: United Nations; WHO: World Health Organization.

The affiliations of HIFA members contributing to the discussion (Figure 4) could be split into several different categories. Nonprofit NGOs were the largest affiliation category and included 77 (31.3%) members of the total. Academia also made up a sizable portion at 57 members (23.2%). The other category contained a number of independent or retired professionals and volunteers.

### Thematic Analysis

In total, 6 major themes, and their subthemes, were identified (Table 1).

**Table 1:** Themes and subthemes identified through the analysis of the COVID-19 discussion on English HIFA^a^ forums.

Theme	Subthemes
Infodemic	Distrust in authority and experts Inconsistent public health messaging Information overload Role of social media Translation needs False health claims
Health system	Handwashing and PPEb Role of CHWsc Ability to test, trace, and conduct surveillance Impact on health care workers Impact on other health services
Digital health literacy	N/A^d^
Economic consequences	N/A
Marginalized peoples	N/A
Mental health	N/A

^a^HIFA: Healthcare Information for All.

^b^PPE: personal protective equipment.

^c^CHW: community health worker.

^d^N/A: not applicable.

#### Theme 1: Infodemic

By far, a significant amount of discussion in the HIFA forums about COVID-19 was regarding the infodemic surrounding it. Specifically, there was considerable input about the spread of misinformation through different mediums, its downstream effects, information gaps, and needs. The importance of making verified health care information accessible to all to prevent infodemics was a common consensus of the HIFA COVID-19 discussion, which is in line with HIFA’s mission. Further, members noted that information that is filtered, simplified, and succinct must be provided through multiple mediums as access to technology can be a barrier. The right information must be presented through the right medium to the right people at the right time.

This theme includes the following subthemes: distrust in authority and experts, inconsistent public health messaging, information overload, the role of social media, translation needs, and false health claims.

##### Distrust in Authority and Experts

A common factor that seemed to drive the infodemic and its impact on the management of the COVID-19 pandemic seemed to be distrust in authority and experts. According to members, many examples of misinformation they have seen circulate online and among their circles questioned the origins of COVID-19. These examples include claims that COVID-19 is a biological weapon, that it was made to sell medicines, or that it was part of a larger global vaccination conspiracy. A few members were concerned that such claims led to distrust in health workers, which has fueled attacks targeting them. In some countries, COVID-19 was seen as a disease of the wealthy and of immigrants due to its association with foreign travel, which has led to instances of racism and xenophobia.

Members also discussed the politicization of the COVID-19 pandemic. Some felt that their respective governments were not being transparent regarding the public health guidance they were providing or about the protocols they had put in place. The accuracy of the number of infections being projected and reported was also questioned. For example, many members questioned the validity of the UK public health officials claiming that 80% of their population could be infected.

Finally, frustration was expressed with how the United States was handling the pandemic. There was discussion that at a time when all governments should be working together, the US government’s threats to pull funding from WHO was not helpful. Here are a few selected posts:

Quite rightly, the [g]overnment is being called to account. All health policy, and especially health policy in public health emergencies such as coronavirus, must be evidence-informed.

Five months into the COVID-19 [p]andemic with daily briefings by [n]ational [g]overnments of African countries, like Nigeria, there is still widespread ignorance amongst the population about COVID-19 and whether it exists at all. Many felt that it (Covid19) is a “scam” by their government “to make money through new drugs and vaccination” (Anecdotal information)!

##### Inconsistent Public Health Messaging

Among the members’ posts, there was general frustration regarding the inconsistency of the public health guidelines being provided. Many were unhappy that some countries were following the guidelines set by WHO, while others were not. Within individual countries, there seemed to be inconsistency in the messaging provided at various levels of government, such as between central and regional, as well as other institutions, such as workplaces and schools. Mass media apparently had also given out contradictory and inconsistent advice. A few members also pointed out that the evolution of public health messaging over time made it difficult to distinguish what the most recent guidelines and protocols were.

To combat this, members suggested consistent, evidence-based guidelines should be given out by all sources, including governments, NGOs, mass media, health care organizations, and individual officials. For this to happen, some supported introducing legislation to hold all these entities accountable in the interest of public health. Here is a selected post:

It is notable that the UK and US (and China) are giving different advice to the general public about what they should do if they develop symptoms and have [a] recent history of travel to affected countries. It's unclear why this is so. The [g]lobal advice on the WHO website indicates…With globalisation of social media among citizens worldwide, it seems important that governments provide the same advice unless there are special contextual reasons why this should not be the case (in which case such reasons should be explicit).

##### Information Overload

The prevalence of too much information about COVID-19 was an issue raised by many members. Information overload was a major factor contributing to the infodemic, as an excess of information makes it difficult to distinguish between what is accurate and what is not. Some members described that this was an issue for everyone, including those who were health literate, since, in some cases, false information was shared and amplified because health professionals themselves were unable to assess its source and accuracy. Many expressed concerns about how information overload overwhelmed the general public, leading to fatigue and a failure to discern the latest guidance.

The rapidly changing status of the pandemic as well as the onslaught of new evidence and research were brought up as some of the causes of the information overload. Moreover, there was duplication of information from multiple organizations attempting to provide knowledge and language translations.

The implementation of a universal and dynamic access point with the latest research, evidence, and guidance to coordinate the influx of information from all sources was put forward by members. Some believed that all sources need to be filtered for misinformation even at the risk of losing knowledge. Here are a few selected posts:

An international website should be created with the majority of languages, containing all the information on the virus, preventive measures, news of its spread and means, international efforts to fight against.

The real problem…is the increase of misinformation, fake news, etc. Every attention and effort should be directed at culling and eliminating misinformation wherever and however it emerges. And as quickly as possible, even at the risk of too much information.

##### Role of Social Media

The role of social media during the COVID-19 pandemic received significant discussion. Social media, including WhatsApp, Facebook, Twitter, and YouTube, were believed by many members to have amplified the spread of misinformation and the infodemic. Concerns were expressed that social media companies were failing to carry out due diligence in filtering misinformation, because they were profiting from the increased engagement with their platforms. A call for companies to be held accountable was raised in a number of posts. An example was shared in which members noticed a significant drop in WhatsApp messages after the South African government threatened legal consequences for anyone engaging in and spreading misinformation on social media.

There was also a discussion that the onus to prevent misinformation should not solely lie with social media companies. Members felt that social media simply was a platform to amplify misinformation that already existed due to the lack of a proper and verified information channel for all to easily access. Thus, arguments were made that social media could be used as a tool to make accurate and verified information accessible.

Finally, the lack of health privacy on social media was a concern because identifying information about individuals who have tested positive for SARS-Cov-2 or were symptomatic was shared in their communities, thereby alienating them. Here is a selected post:

Misinformation has played a major role in worsening the situation across the world in its rapid response to the Covid-19 creating a state of widespread panic especially with readily available access to social media as compared to a decade ago. Although this could be beneficial in many ways, it is being misused time and again to spread conspiracy theories and other forms of misinformation about the Covid-19.

##### Translation Needs

Throughout the English HIFA thread on COVID-19, there were multiple requests for the rapid translation of current guidelines and resources to other languages and dialects. Members reported that automatic language translation tools, such as Google Translate, were not accurate and did not contain regional dialects. Additionally, misinformation was also prevalent in lesser known languages and dialects and it was not being addressed. General public health advice given out by international organizations, such as WHO, may not be applicable to local settings or consistent with local regulations, and so there was a need for contextualization.

Finally, some pointed out that governments and public health organizations were indirectly excluding foreigners, such as tourists and expats, by not providing local advice and guidance in languages other than the country’s official ones. Here is a selected post:

it is important that evidence-based messages (from the World Health Organization & other reliable sources) are tailor-made in their local languages to reach and empower them.

##### False Health Claims

The prevalence of false health claims regarding how COVID-19 spreads, its treatment, and its prevention was discussed. Although most of these claims did not pose a danger, some directly contradicted official public health and medical advice, such as gathering in places of worship and taking unproven medications. Religious prophets and self-appointed “experts” in LMICs were identified as primary promoters of such false information, although false claims have been made in many high-income countries.

The misuse of chloroquine as a medication to treat COVID-19 was a major topic of discussion. Members were frustrated that influential political leaders, news media, and medical professionals were endorsing chloroquine to be an effective medication for COVID-19 without verified evidence. Some members noticed that physicians and pharmacists in their regions have started to prescribe chloroquine to patients, causing shortages and, in some cases, deadly side effects. Here are a few selected posts:

With this outbreak I worry about Nigeria for the reason that already there are “prophets” with claims they can cure coronavirus and others are selling ANOINTED SOAP to prevent contracting the virus.

This is probably the most shocking and most unethical practice I have heard of related to corona. How can a politician and a businessman dictate such medical practices? How can health personnel (doctors and pharmacists) allow this to happen for themselves and their families.

#### Theme 2: Health System

The ability of health systems to handle COVID-19 was another theme that emerged from the forums. This theme includes discussion about handwashing and personal protective equipment (PPE), the role of CHWs, the ability to conduct surveillance for COVID-19, and the impact on health care workers and other health services.

##### Handwashing and Personal Protective Equipment

Members expressed concern about the reduced supply of PPE in both LMICs as well as in areas of the health care system outside of hospitals, such as long-term care homes. Suggested alternatives included cloth masks, reusable visors, and even steam inhalations as being better than nothing regardless of a lack of evidence of their efficacy. Government budgetary decisions were questioned as some members felt that public money should be spent toward acquiring critical health equipment over other areas. The lack of hand sanitizers and clean water in some regions had apparently made it difficult to follow WHO’s advice on frequent handwashing. For this, an alternative solution of washing hands with ash was brought up. Here is a selected post:

We experienced a very severe and unjustifiable lack of protection devices for nurses and doctors: a severe lack of masks (all of them), a severe lack of vital supporting devices and many other criticalities.

##### Role of Community Health Workers

CHWs were seen as essential for addressing the COVID-19 pandemic. Their role included making home visits to persons under suspicion of having COVID-19, thereby reducing unnecessary exposure to others and triaging them to more advanced care, if needed. Furthermore, CHWs can educate the local communities they are part of, address any misinformation, and help conduct surveillance of cases. Here is a selected post:

CHWs promoted pandemic preparedness prior to the epidemics by increasing the access to health services and products within communities, communicating health concepts in a culturally appropriate fashion, and reducing the burdens felt by formal [health care] systems. During the epidemics, CHWs promoted pandemic preparedness by acting as community-level educators and mobilizers, contributing to surveillance systems, and filling health service gaps.

##### Ability to Test, Trace, and Conduct Surveillance

There was discussion and concern around some countries’ ability to test, trace, and conduct surveillance. The limited number of testing kits and surveillance systems in African countries led to a number of unaccounted-for infections. Emphasis was placed on the importance of being proactive and taking a strict approach to travel restrictions and isolation even before COVID-19 became a considerable threat in such countries. Some suggested that certain African countries, such as Nigeria, may be better prepared due to their prior/continuing experience with Ebola, HIV/AIDS, tuberculosis, and other recent epidemics. Here is a selected post:

That surge in cases is causing deep unease in countries like Kenya, which have strong commercial ties to China, but, like many other developing nations, have only limited health and surveillance systems...At the moment, Kenyan hospitals would be unable to confirm whether someone has been infected as they do not have the “reagent kits” necessary to identify the coronavirus, officially designated 2019-nCoV.

##### Impact on Health Care Workers

The negative treatment of health care workers during COVID-19 and how it should be addressed arose in this theme. Experiences from Italy during the height of the epidemic were shared, showing instances of health care workers’ physical and mental exhaustion. Similarly, it was shared that many health care workers were unprepared to make difficult triage decisions regarding who should be allocated valuable and limited health care resources, such as beds in intensive care units (ICUs). Increased instances of violence, abuse, and discrimination toward health care workers were reported.

Members mentioned that some occupations that make frequent contact with persons with COVID-19 were not being supported the same way as doctors and nurses were despite having an above-average risk of contracting the disease—specifically, allied health occupations, such as pharmacists and physiotherapists, as well as admin staff and hospital caretakers. Finally, the importance of addressing the SEISMIC (Skills, Equipment, Information, Systems support, Medicines, Incentives, Communication) needs of health care workers was brought up. Here is a selected post:

I am looking at the *self care* of front line workers working for COVID-19 prevention. We need practicable measures for the front line workers within their current working conditions and my guidelines must be seen in that context.

##### Impact on Other Health Services

The impact of COVID-19 on other health services generated discussion as well. Specifically, access to palliative care, cancer care, and reproductive and women’s health, including the use of birth control, provision of abortions, HIV testing, and addressing of gender-based violence, were brought up. Here is a selected post:

I am increasingly concerned that the national response to the pandemic will (in some countries, at some stages in the evolution of the pandemic) have an even greater negative impact on health than the virus itself…Birth control, GBV-support, and HIV testing are out of reach to more women as COVID-19 shutters clinics around the globe…The closures are making it difficult for millions of women to access contraception, abortions, HIV testing, or support for gender-based violence.

#### Theme 3: Digital Health Literacy

Discussion on digital health literacy included access to technology/internet services and dissemination of information through alternative and innovative media. The lack of access to adequate internet services, especially in conflict-prone places with internet shutdowns and slow connectivity, presented barriers to the COVID-19 response. A few members also pointed out that censorship was imposed on news websites by several governments. Additionally, there was concern that in places such as India and Nigeria, reduced smartphone availability and internet penetration excluded many from access to online health care information.

Members iterated that unequal access to adequate health care information and COVID-19 guidelines online posed a gap that could potentially be fulfilled by the utilization of radio, posters, and television broadcasts. An innovative solution was introduced through highlighting the work of the Bangladesh NGO Network for Radio and Communication (BNNRC), which disseminates information to internet deserts through an innovative network of radio broadcasters. Here are a few selected posts:

In Nigeria and most of Africa, smartphone and internet penetration varies between 20 [and] 40% in different areas…due to this a large number of the population is excluded from access to online health care information.

In the response to COVID-19, we see how vital it is to get accurate and trusted messages to people so that they know what they need to do and where they can get help when they need it. Now 18 Community Radios stations in Bangladesh have been broadcasting 165 hours of [c]oronavirus prevention education with the active participation of community people. There are 1000 community youth and youth women community radio broadcasters broadcast programs for 6.5 million listeners and viewers.

#### Theme 4: Economic Consequences

Discussion regarding the economic consequences of the pandemic and resulting lockdown was another emergent theme. Various members shared their experiences and opinions highlighting challenges being faced and solutions or actions in implementation. Specifically, members deliberated about the economic sustainability of a lockdown in LMICs, the inability to meet basic needs leading to increased poverty-related deaths, and the importance of government relief and stimulus. Here is a selected post:

For regions like [s]ub-Saharan Africa, COVID-19 can be a perfect storm in the form of a health problem, and above all, an economic catastrophe for which they lack a safety net…I could think that although these people do not want to be exposed to the virus, it is a population that must continue working to survive, unless the government does something about it.

#### Theme 5: Marginalized Peoples

The impact of COVID-19 on marginalized communities focused particularly on the impact on slums in India and Nigeria, the favelas in Brazil, people experiencing homelessness, immigrants, refugees, and those at risk for severe manifestations of the disease. Furthermore, members raised concerns that the public health advice being provided was not helpful for these communities, as it may be impossible for them to follow (eg, social distancing in overcrowded shelters and slums). Here is a selected post:

Yes, what is the minimum distance? The overcrowding is unavoidable in my environment…I know some homes and settlements in my environment are more crowded than the schools. They live in slums.

#### Theme 6: Mental Health

The impact of COVID-19 on mental health included topics centered around the mental health of vulnerable populations and addressing fear, anxiety, and psychological stress stemming directly or indirectly from COVID-19. A few members shared their personal struggles with mental health. Here is a selected post:

India is currently under [lockdown] to reduce the risk of coronavirus infection. The plight of senior citizens has become pitiable. I would, if there are any organizations in India or other countries, who can speak to them to alleviate their depression.

### Timeline Analysis

**Figure 5:**
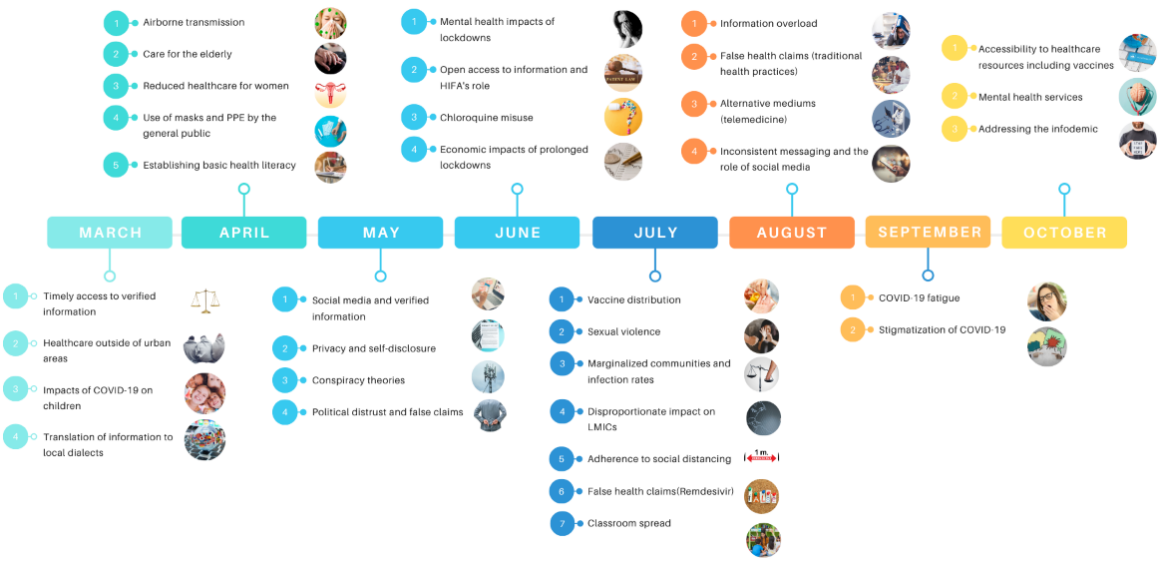
Timeline analysis of the HIFA COVID-19 discussion highlighting major topics from March to October 2020. HIFA: Healthcare Information for All; LMIC: low- and middle-income country; PPE: personal protective equipment.

The timeline analysis (Figure 5) identified topics of discussion surrounding COVID-19 on the English HIFA forums from March 2020 to October 2020. From the timeline analysis, discussion during the earlier months was centered around access to verified health information, translation of public health guidelines, understanding what can be done to prevent the spread of COVID-19, and the preparedness of different health systems. Discussion around the prevalence of the infodemic and misinformation took place mostly during May and June 2020. The end of June going into July 2020 saw the discussion focused on the impacts of a lockdown, including its economic consequences, its effects on marginalized communities, and its toll on mental health. Discussion during August and September 2020 revolved around COVID-19 fatigue and changing public health guidelines amid a second wave. Finally, vaccine production, distribution and administration as well as addressing the infodemic were discussed in October 2020.

## Discussion

### Principal Findings

We present 8 months of spontaneous discussion relating to COVID-19 on English HIFA forums. Themes included the infodemic, health system, digital health literacy, economic consequences, marginalized peoples, and mental health. The infodemic and related issues of access to reliable health care information and misinformation were the predominant topic.

### Infodemic and Related Issues

An infodemic, as defined by WHO, is an overload of information, some reliable and some unreliable [20]. Never have we all been so aware of the importance of reliable health care information and yet so vulnerable to misinformation. The central problem is that the general population is unable to differentiate between reliable and unreliable information. This is not new: it has always been the case that unreliable information has misled people, with disastrous consequences. For example, the widespread belief that one should stop giving fluids to a child with diarrhea is 1 of hundreds of examples. More recently, the Ebola outbreak was associated with an infodemic [21]. However, the current infodemic relating to COVID-19 is far worse. What has changed is that increasingly more people are vulnerable to misinformation on social media [22], which propagates false information much more readily than true information. Increased connectivity has paradoxically worsened access to reliable health care information.

Contributing factors include public distrust of the authorities that are responsible for public health messaging, leading to conspiracy theories and denial of the existence of COVID-19. We have seen how public health messaging is partly to blame. Communication with the public may be ineffective due to inappropriate content and format, changing messages as the pandemic unfolds, and inconsistency of messaging. In some countries, politicization drives misinformation; in the United States, for example, vaccine refusal is strongly associated with Republican voters.

### Implications for Policy and Practice

A fresh and important perspective was brought by the participants in this discussion, namely the central importance of facilitating access to reliable health care information as a vital aspect of protecting people from misinformation. Increasing people's access to the internet alone will not help and may make things worse. The key is to help people differentiate between reliable and unreliable health care information. One approach is to increase health literacy, but we have noted in our discussions that even WHO staff are vulnerable to misinformation. Although health literacy is important, new approaches are needed to help people differentiate reliable from unreliable information. The Health on the Net Foundation has led the way in certifying websites that have robust methods of ensuring reliability, but few people are aware of it. Recently, a case was made for WHO to steward a new top-level health domain for reliable health care information [23], but this failed in favor of commercial forces. Better solutions are needed to ensure that every person has access to the reliable health care information they need to protect their own health and the health of others.

### Future Research

Future research should explore the role of various approaches to helping people differentiate between reliable and unreliable information, drawing on mixed methods, such as systematic review and consultations. Furthermore, emerging research surrounding the COVID-19 infodemic has demonstrated a correlation between susceptibility to misinformation and both vaccine hesitancy and a reduced likelihood to comply with health guidance measures [24]. As such, interventions that aim to improve critical thinking and trust in science may be a promising avenue for future research with regard to addressing infodemics and their downstream consequences.

### Strengths and Limitations

One major strength of this analysis is that it brings forth several perspectives of the global COVID-19 response from study participants spanning many geographical regions, professions, and affiliations. The themes that have emerged from this analysis highlight personal recounts, reflections, suggestions, and evidence around dealing with COVID-19-related misinformation. The timeline also provides additional pointers on how discussions surrounding COVID-19 evolved and help to understand the shift in focus across themes and topics that took place. However, this information must be interpreted with caution and cannot be generalized as a global exchange of discussions on COVID-19.

One limitation is that this analysis does not present any novel information or findings. Furthermore, as many of the study participants are from a public health, health policy, or related background, certain views and opinions are overexpressed.

### Conclusion

This qualitative analysis study highlights the major themes that emerged from the discussions surrounding COVID-19 on the multidisciplinary HIFA forums and can help to understand the type of information needs that arose during the pandemic. The timeline analysis from this study highlights how discussions surrounding the COVID-19 pandemic evolved and when the various themes took place. The perspectives identified provide a multilateral insight into what can contribute to infodemics and enable the development of solutions to manage both the current and future infodemics.

This study used an observational method to understand the themes and perspectives surrounding the evolving COVID-19 pandemic shared in an online multidisciplinary global health forum with a focus on misinformation, information needs, and regional impacts. The results show that the discussion was rich and had representation from multiple disciplines and geographical locations. Many members shared common concerns and frustrations regarding the ensuing infodemic, with the consensus being that all public health organizations and institutions must effectively anticipate and address infodemics in the future to achieve maximal public adherence to guidelines and mitigate danger. Multiple approaches must be used, including holding influential figures and mass media accountable, deploying rapid knowledge and language translation efforts, using multiple channels of communication to disseminate information, and, most importantly, making verified health care information accessible. As such, HIFA stands in solidarity with WHO in its call to action to distribute the right message at the right time from the right messenger through the right medium.

## Abbreviations

CHW: community health worker
HIFA: Healthcare Information for All
LMIC: low- and middle-income country
NGO: nongovernment organization
PPE: personal protective equipment
UN: United Nations
WHO: World Health Organization
